# The Subarachnoid Hemorrhage–Weather Myth: A Long-Term Big Data and Deep Learning Analysis

**DOI:** 10.3389/fneur.2021.653483

**Published:** 2021-05-05

**Authors:** Moritz Helsper, Aashish Agarwal, Ahmet Aker, Annika Herten, Marvin Darkwah-Oppong, Oliver Gembruch, Cornelius Deuschl, Michael Forsting, Philipp Dammann, Daniela Pierscianek, Ramazan Jabbarli, Ulrich Sure, Karsten Henning Wrede

**Affiliations:** ^1^Department of Neurosurgery and Spine Surgery, University Hospital Essen, University of Duisburg-Essen, Essen, Germany; ^2^Department of Computer Science and Applied Cognitive Science, University of Duisburg-Essen, Duisburg, Germany; ^3^Institute of Diagnostic and Interventional Radiology and Neuroradiology, University of Duisburg-Essen, Essen, Germany

**Keywords:** subarachnoid hemorrhage-weather, SAH, hemorrhagic stroke, big-data, deep-learning, subarachanoid hemorrhage, machine learning

## Abstract

**Objective:** The frequency of aneurysmal subarachnoid hemorrhage (aSAH) presents complex fluctuations that have been attributed to weather and climate changes in the past. In the present long-term big data and deep learning analysis, we have addressed this long-held myth.

**Methods:** Bleeding dates and basic demographic data for all consecutive patients (*n* = 1,271) admitted to our vascular center for treatment of aSAH between January 2003 and May 2020 (6,334 days) were collected from our continuously maintained database. The meteorological data of the local weather station, including 13 different weather and climate parameters, were retrieved from Germany's National Meteorological Service for the same period. Six different deep learning models were programmed using the Keras framework and were trained for aSAH event prediction with meteorological data from January 2003 to June 2017, with 10% of this dataset applied for data validation and model improvement. The dataset from July 2017 to May 2020 was tested for aSAH event prediction accuracy for all six models using the area under the receiver operating characteristic curve (AUROC) as the metric.

**Results:** The study group comprised of 422 (33.2%) male and 849 (66.8%) female patients with an average age of 55 ± 14 years. None of the models showed an AUROC larger than 60.2. From the presented data, the influence of weather and climate on the occurrence of aSAH events is extremely unlikely.

**Conclusion:** The myth of special weather conditions influencing the frequency of aSAH is disenchanted by this long-term big data and deep learning analysis.

## Introduction

Aneurysmal subarachnoid hemorrhage (aSAH) is a common cause of stroke with high mortality and morbidity. The worldwide annual incidence of aSAH is 7.9 per 100,000 person-years (1). The worldwide annual death toll of aSAH is approximately half a million people. The highest prevalence is in the age group of 35 to 60 years, and thus half of the patients affected are under 50 years of age (2). Known risk factors of cerebral aneurysms include smoking, arterial hypertension, congenital disorders of the connective tissues (e.g., “Marfans syndrome” or “Ehlers–Danlos syndrome”), positive family history of the disease, age (>40 years) as well as gender (male to female ratio: 2/3). Further risk factors are alcohol and drug abuse (particularly cocaine), polycystic kidney disease, and fibromuscular dysplasia (3, 4). Several scores such as “UIATS”, “ELAPSS,” or “PHASES” have been created to predict aneurysm growth and the rates of aSAH using those risk (5–7). However, numerous authors have attributed weather and climate to influence the occurrence of aSAH events (8–22). Unlike for the clear correlation between myocardial infarction and cold weather (23), the results of research on the incidence of aSAH succeeding weather and/or climate changes have been inconsistent. Over the last decades, starting in the 1980s (16), no general agreement could be established, and the persistent myth of “aneurysm weather” perseveres. Different study cohorts, geographical areas, study designs, and statistical methods have led to contradicting results. Computing power and the amount of research in big data analysis have been constantly increasing throughout the last decades. Deep learning data analysis has made its way into medical mainstream. The availability of large datasets allows event predictions, pattern recognition, and detailed image analysis. Consequently, these networks can aid clinicians for diagnosis and treatment and can, therefore, improve the quality of patient care (24). Several clinical applications have been reported in the last years, including analysis of electrocardiograms (25), diagnosis of pneumonia in chest X-rays (26), and virtual contrast application in cranial magnetic resonance imaging (27). In the field of neurosurgery, machine learning networks were used to predict the postoperative mortality rates of patients with spinal metastases (28). This study aimed to analyze a large range of weather and climate parameters and their effect on the occurrence of aSAH. The big data and deep learning approach allowed to the simultaneous analysis of 13 different weather and climate parameters and 1,271 aSAH events over a course of 6,334 days (83,613 data points).

The results are reported in accordance with the Strengthening the Reporting of Observational Studies in Epidemiology (STROBE) guidelines for reporting observational studies.

## Methods

The University of Duisburg-Essen ethical committee authorized the study (registration number: 15-6331-BO), and all patients or their relatives provided written informed consent. The study was conducted according to the principles of the Declaration of Helsinki and was compliant with the Health Insurance Portability and Accountability Act.

### Study Area and Inclusion Criteria

The neurovascular department at the University Hospital of Essen is one of the most frequented referral centers for patients with aneurysmal SAH in the Ruhr area. The region is densely populated with over five million inhabitants, located close to Germany's western border with the Netherlands. The climate in the area is moderate with four distinct seasons. The climate throughout the whole regions tends to not vary significantly due to the absence of large mountain ranges or other geographical weather disturbances. Data from 1,271 patients admitted to our neurovascular department for the treatment of aSAH between January 2003 and May 2020 were collected from our continuously maintained prospective database. Evaluation included bleeding dates, basic demographic data as well as 13 weather parameters for each day (6,334 days). The inclusion criteria were (a) radiographic verification of the bleeding source of aSAH (by digital subtraction angiography and/or computed tomography angiography) and (b) information about the exact date of the ictus.

### Meteorological Data

Meteorological data was obtained from the closest weather station (Bredeney #1303), approximately 3 km southwest of our department. It was kindly provided by the Climate Data Center of the German meteorological service (Deutscher Wetterdienst). The following weather parameters were included: daily maximum wind speed (m/s), daily mean wind speed (m/s), daily amount of rainfall in (mm), daily amount of sunshine (hours), daily amount of snowfall (cm), mean daily vapor pressure (hPa), mean daily atmospheric pressure (hPa), mean daily humidity (%), mean daily temperature (°C), maximum and minimum daily temperature at 2 m above ground level (°C), minimum daily temperature at 5 cm aboveground (°C), and cloud coverage.

### Data Preprocessing

Various techniques were applied to preprocess the data, including imputation of missing values using scikit-learn (29) and quantile-based discretization to smoothen the data by identifying outliers (30) using the Pandas software library (31). Standardization and Gaussian distribution with zero mean and unit variance were achieved with the scikit-learn library (29). Feature engineering allowed modeling the impact of changes in temperature from the previous day's maximum to the minimum of the next day as a trigger for SAH. The chi-square test was applied to determine significant associations between features (weather parameter) and class labels (ictus/no ictus). Pearson correlation coefficient was calculated to identify the correlation of different meteorological factors on admission days for aSAH.

### Training, Validation, and Test Split

The meteorological dataset from January 2003 to June 2017 was used as training data, and 10% of the training data was selected to validate this data and improve the model. Meteorological data from July 2017 to May 2020 was chosen as test data. Sequence-to-sequence classification with sequence lengths of 5 days (2 days before and after the ictus) allowed the correction for bias from overlapping positive and negative classes.

### Deep Learning Models

We used pattern recognition algorithms in six standard deep learning models that were implemented with the Keras framework for Python (32), Dense Autoencoder, bi-directional long short-term memory (Bi-LSTM), standard long short-term memory (LSTM), LSTM autoencoder, Dense (feed forward neural network), convolutional neural network (CNN), and CNN+LSTM.

### One-Class Classification

In the context of neural networks, one-class classification is also called a “novelty detection technique.” It has been implemented extensively in the field of anomaly detection to classify rare events in cases with a large class imbalance. This technique considers either positive or negative instances instead of distinguishing between two classes. Autoencoders used for this purpose are trained to perform auto-associative mapping, that is, identity function. The classification is made based on a reconstruction error between the input and predicted output patterns, for example, Euclidean, Mahalanobis distance, absolute error, or the squared sum of errors. We used dense and LSTM-based autoencoders (33–35). In this type of classification, the sequence of information is not relevant. Therefore, features are extracted either from days with a bleeding event (in case of positive) or days without a bleeding event (in case of negative).

### Two-Class Classification

In contrast to the previous technique, sequence-to-sequence-based classification models were applied. These models use the current input and also the previous values to calculate the result. Five days preceding each positive and negative day were included, resulting in a sequence of six consecutive days. These sequence-to-sequence models make the judgment based on the entire sequence. We investigated standard LSTM and also Bi-LSTM models (36–38). Bi-LSTM, in contrast to standard LSTM models, read the sequence from left to right and then right to left. Furthermore, we combined LSTM with CNN, which helps to extract more and more detailed features (39).

### Evaluation Metrics and Testing

The overall accuracy, which is the proportion of test examples, is the metric that is most widely used to evaluate a classifier's performance. When a dataset is imbalanced, the accuracy will favor the overrepresented classes. This leads to misclassification. A measure of quality that addresses these issues is the AUROC (area under receiver operator characteristic). We used the AUROC as the main metric to compare the performance of classifiers trained with our datasets. However, for the purpose of evaluation, we also report precision (ratio of correctly predicted positive observations to the total predicted positive observations), recall (ratio of correctly predicted positive observations to all the observations in actual class), and F1 score (harmonic mean of precision and recall) along with the AUROC values.

### Statistical Analysis

Data preprocessing, statistical analysis, and model training was implemented in Python (Version 3.6) (40). The Python libraries utilized for data preprocessing included Numpy (41), Pandas (42), and scikit-learn (29). Model training was implemented in the Keras library (32). Statistical analysis was carried out using the Seaborn (43) and MatplotLib (44) libraries.

Any data not published within the article is available in the public repository “figshare” (https://doi.org/10.6084/m9.figshare.14129960).

## Results

**Figure 1:**
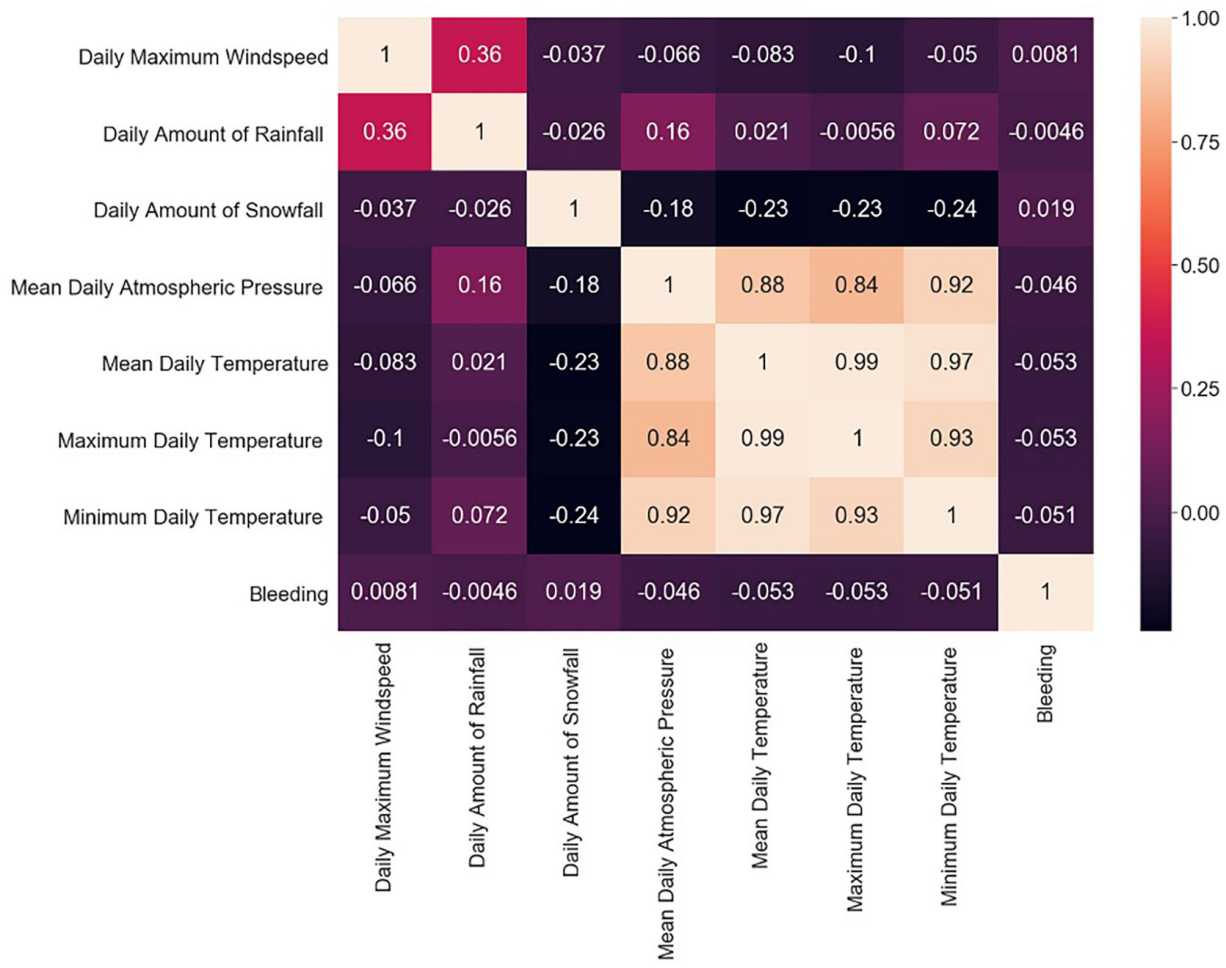
Pearson correlation chart showing the linear correlations between individual weather parameters and bleeding days.

**Figure 2:**
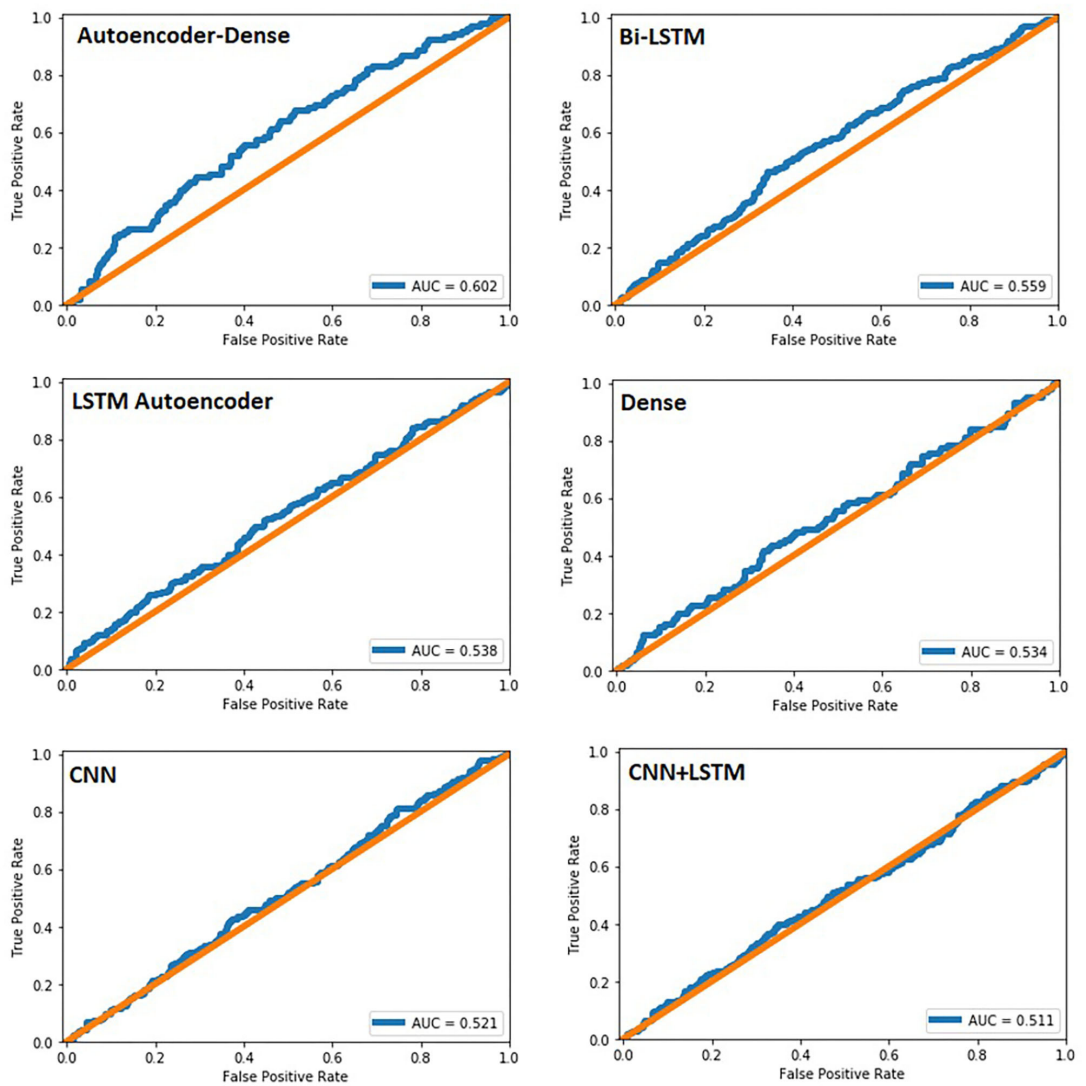
Area under the curve for all six networks created. In our study, it was used to predict bleeding events within our dataset.

The study group comprised 422 (33.2%) male and 849 (66.8%) female patients with an average age of 55 years (range, 19–94; SD ± 14). During data preprocessing, chi-square test (Table 1) showed a significant association between seven out of 14 features and the class label (aSAH), revealing a slight correlation between the data. The Pearson correlation chart (Figure 1) showed only a weak linear correlation within the majority of the chosen meteorological parameters. All networks showed similar, high classification accuracy, represented by Precision, Recall, and F1. However, they were not able to reproduce bleeding days by weather data alone. This is measured by the AUROC. In our scenario, the accuracy of the test depends on how well the test separates bleeding from non-bleeding days using meteorological data. The highest AUROC value was produced by Dense Autoencoder, with a value of 60.2 (Figure 2). LSTM and LSTM+CNN models that take past values and Bi-LSTM model, which also considers future values, also had similar outcomes (Table 2). This provides strong evidence that the incidence of aSAH has no relevant correlation with meteorological factors.

**Table 1:** Results of “chi-squared test” performed during data preprocessing, illustrating association between aSAH and weather features.

**Feature**	***P*-value**	**Correlation**
Daily maximum windspeed (m/s)	0.01584	Yes
Daily mean windspeed (m/s)	0.39967	No
Daily amount of rainfall in (mm)	0.03829	Yes
Daily amount of sunshine (hours)	0.75359	No
Daily amount of snowfall (cm)	0.00098	Yes
Mean daily vapor pressure (hPa)	0.00264	Yes
Mean daily atmospheric pressure (hPa)	0.71198	No
Mean daily humidity (%)	0.82945	No
Maximum daily temperature at 2 m above ground level (°C)	0.00533	Yes
Minimum daily temperature at 2 m above ground level (°C)	0.00544	Yes
TDP for mean daily temperature (°C)	0.14743	No
Mean daily temperature (°C)	0.01411	Yes
Minimum daily temperature at 5 cm above ground (°C)	0.07481	No
TDP for minimum daily temperature at 5 cm above ground	0.20424	No

**Table 2:** Overview of the precision*, recall*, F1*, and AUROC values for all six deep learning models *(weighted average).

	Precision*	Recall*	F1*	AUROC*
Dense Autoencoder	72	62	65	60.2
Bi-LSTM	71	80	72	55.9
LSTM Autoencoder	73	80	75	53.8
Dense	64	80	72	53.4
CNN	69	77	72	52.1
LSTM	67	82	74	50.8

## Discussion

Weather and climate changes have been attributed to influence aSAH events. Several studies using classical statistics have been published in the past decades with contradicting results. To our knowledge, this is the first study using deep-learning analysis utilizing several weather parameters simultaneously. Deep learning models each have specific strengths and weaknesses. Therefore, six different deep learning models were evaluated to find the most suitable solution for our problem with the highest sensitivity and specificity. The largest analysis on the topic to this date is a multicenter, retrospective study based on the admission diagnosis of 155 US hospitals during the calendar years 2004 to 2008 (*N* = 7,758) (21). Analyzing temperature, pressure, and humidity, Cowperthwaite and Burnett found no influence of these parameters on the incidence of aSAH. The biggest limitation of this study is the possible discrepancy between the date of aneurysm rupture and hospital admission. On the other hand, the large nationwide study design allowed for the correction of local biases. Several single-center studies found a correlation of aSAH events and environmental pressure, low temperatures, and sudden temperature change. Van Donkelaar et al. investigated the influence of environmental pressure changes on aneurysm rupture. They reported that an increase in pressure on the second and third days before ictus was correlated with a higher incidence of aSAH. They assumed that pressure changes were a delayed trigger of aSAH (18). Several other studies supported these findings (8, 17). Analyzing sequences of 5 days before the ictus allowed us to detect the possible influence of even very small weather changes. Neither pressure changes nor any other parameters were identified as a trigger of aSAH. In accordance with our results, Landers *et al*. did not find any association between aSAH and an increase in barometric pressure either (22). Other authors reported sudden temperature changes to be a relevant risk factor for aSAH. Gill et al. state that a 1°F temperature drop from 1 day to the next is associated with 0.6% increased risk of aSAH (12). This subtle increase is most likely explained by selection bias and the relatively small study cohort. Backes et al. matched 18,714 patients from the Dutch SAH registry with corresponding ICD codes to the average weekly temperature and found low temperatures to be connected with an increase in aSAH frequency (9). These results support a seasonal dependency of aSAH incidence that we did not find in our cohort. On the other hand, Rivera-Lara et al. found an increase in aSAH incidence also on warm days, but only when the temperature significantly dropped within that same day (15). Their results were supported by two other research groups (19, 45). Our data does not support these findings, and from our experience, it remains questionable if it is possible to determine the time of the ictus with the required accuracy, especially in retrospective datasets. Muroi et al. showed a seasonal variation only in patients younger than 59 in their prospective study with 489 patients from Zurich (14). Chyatte et al. described relevant seasonal fluctuations as well, with a peak in spring for both men and women in their single-center study from the United States (10). In contrast, there were no seasonal variations in hospital admissions in our patient cohort. On the one hand, this could be explained by the moderate central European climate in western Germany, with only subtle weather changes from day to day, but from our analysis it is more likely that the weather and climate changes have no relevant influence on the frequency of aSAH. In summary, previous research on the influence of weather and climate changes on the frequency of aSAH yielded contradictory results. During the data preprocessing stage of the present study, ictus dates and certain weather parameters showed some correlation. However, the applied deep-learning models were neither able to reproduce or predict bleeding days nor able to detect a pattern in the influence of weather and climate as a whole or individually for each parameter.

There are some limitations to our study. The analysis is based on a prospective aSAH dataset starting in 2003. Individual habits and social and working conditions that have not been assessed might have changed during the observation period and therefore bias the analysis. The presented results are only valid for moderate weather and climate conditions like the ones prevalent in central Europe. More extreme conditions could potentially lead to different results. Patients who died before admission or patients who were admitted at surrounding neurosurgical departments were not included within the analyzed dataset.

## Conclusion

The myth of special weather and climate conditions influencing the frequency of aSAH is disenchanted by this long-term big data and deep learning analysis. After all, the weather does not appear to influence the risk of aneurysm rupture.

## Data Availability Statement

The original contributions presented in the study are included in the article/supplementary material, further inquiries can be directed to the corresponding author/s.

## Ethics Statement

The studies involving human participants were reviewed and approved by The University of Duisburg-Essen ethical committee (Registration number: 15-6331-BO). The patients/participants provided their written informed consent to participate in this study.

## Author Contributions

MH designed and conceptualized the study, analyzed the data, and drafted the manuscript for intellectual content. AAg performed data analysis and created the deep learning networks. AAk performed data analysis and supervised the creation of the deep learning networks. AH and MDO had a major role in the acquisition of data. OG, CD, MF, PD, DP, and US drafted and revised the manuscript for content, and including medical writing for content. RJ had a major role in the acquisition of data and drafting/revision of the manuscript for content, including medical writing for content. KHW contributed to conceptualization, methodology, and writing—original draft and final approval of the version to be published. All authors contributed to the article and approved the submitted version.

## Conflict of Interest

The authors declare that the research was conducted in the absence of any commercial or financial relationships that could be construed as a potential conflict of interest.

## Abbreviations

CT: computed tomography
CTA: computed tomography angiography
MRA: magnetic resonance angiography
DSA: digital subtraction angiography
SAH: subarachnoid hemorrhage
aSAH: aneurysmal subarachnoid hemorrhage
AUROC: area under the receiver operating characteristics
ROC: receiver operating characteristic
LSTM: long short term memory
Bi-LSTM: directional long short term memory
CNN: convolutional neural network
ICD: International Classification of Diseases.
